# Meeting demand for family planning within a generation: prospects and implications at country level

**DOI:** 10.3402/gha.v8.29734

**Published:** 2015-11-09

**Authors:** Yoonjoung Choi, Madeleine Short Fabic, Sennen Hounton, Desmond Koroma

**Affiliations:** 1 United States Agency for International Development, Washington, DC, USA; 2 United Nations Population Fund, New York, NY, USA

**Keywords:** demand for family planning, modern contraception, Sustainable Development Goals

## Abstract

**Background:**

In order to track progress towards the target of universal access to sexual and reproductive health care services of the post-2015 Sustainable Development Goals (SDGs), a measure (demand for family planning satisfied with modern contraceptive methods) and a benchmark (at least 75% by 2030 in all countries) have been recommended.

**Objectives:**

The goal of this study was to assess the prospects of reaching the benchmark at the country level. Such information can facilitate strategic planning, including resource allocation at global and country levels.

**Design:**

We selected 63 countries based on their status as least developed according to the United Nations or as a priority country in global family planning initiatives. Using United Nations estimates and projections of family planning indicators between 1970 and 2030, we calculated percent demand for family planning satisfied with modern contraceptive methods for each year and country. We then calculated the annual percentage point changes between 2014 and 2030 required to meet the benchmark. The required rates of change were compared to current projections as well as estimates between 1970 and 2010.

**Results:**

To reach the benchmark on average across the 63 countries, demand satisfied with modern methods must increase by 2.2 percentage points per year between 2014 and 2030 – more than double current projections. Between 1970 and 2010, such rapid progress was observed in 24 study countries but typically spanning 5–10 years. At currently projected rates, only 9 of the 63 study countries will reach the benchmark. Meanwhile, the gap between projected and required changes is largest in the Central and West African regions, 0.9 and 3.0 percentage points per year, respectively. If the benchmark is achieved, 334 million women across the study countries will use a modern contraceptive method in 2030, compared to 226 million women in 2014.

**Conclusions:**

In order to achieve the component of the SDGs calling for universal access to sexual and reproductive health services, substantial effort is needed to accelerate rates of progress by a factor of 2 in most study countries and by a factor of 3 in Central and West African countries.

**Paper context**

Achieving universal access to sexual and reproductive health care services is a target of the post-2015 Sustainable Development Goals (SDGs). To monitor progress, a measure (demand for family planning satisfied with modern contraceptive methods) and a benchmark (at least 75% by 2030 in all countries) have been recommended. This paper assesses the prospects of reaching the benchmark at the national level in 63 selected countries. Study results show that substantial effort is needed to accelerate rates of progress, by a factor of 2 in most study countries, suggesting a need for strategic planning, including resource allocation, at the global and country levels.

Expanding access to family planning has been a key objective of health and development programmes for decades. The 2012 London Summit on Family Planning and other global and regional initiatives brought renewed emphasis on family planning's importance to health (1–3), economic wellbeing (4), empowerment, and environment (5). Accelerating family planning's progress is again a driving aim of the international community and many individual countries.

Aligning with global and local initiatives and evidence, the Sustainable Development Goals (SDGs) include the target of ‘universal access to sexual and reproductive healthcare services, including for family planning, information and education, and the integration of reproductive health into national strategies and programmes’ (6). To track the progress of this target, a measure (demand for family planning met with modern contraceptive methods) and a benchmark (at least 75% by 2030 in all countries) have been suggested (7) and have received positive feedback from the international family planning community and beyond. Recently, the United Nations Technical Support Team to the General Assembly Open Working Group on the SDGs recommended the indicator, and scores of national statistics organisations described it as ‘highly relevant’ to the SDGs (8).

The measure reflects the aim of family planning programmes – to support the rights of individuals and couples to choose whether and when to have a child by providing them with effective means to implement their decisions – and promotes voluntarism, informed choice, rights, and equity (7, 9). The proposed benchmark aligns with current levels of demand for family planning being satisfied in most developed countries and for various subpopulations in developing countries, as well as with historical experiences of formerly low-income countries (7). The benchmark is also ambitious: it will only be achieved if progress towards meeting demand for family planning is accelerated, especially in low-income countries.

It is critical to understand the progress that will be required on a country-by-country basis to best direct resources and guide strategic planning. Our study aims to understand prospects of meeting the benchmark by 2030 using data from 63 selected countries. Specific objectives were as follows: 1) to estimate the progress needed to meet the benchmark (i.e. at least 75% of demand for family planning satisfied with modern contraceptive methods) in each country and 2) to compare the required progress to current projections by United Nations as well as historic experience. Further, in order to illustrate various prospects across countries, we examine the data in detail for three countries: Burkina Faso, Ethiopia, and Nigeria.

## Methods

### Study countries

We selected 63 countries for the study to understand country-level prospects. These countries are either among the least developed countries, according to the United Nations classification (10) or priority countries for international family planning initiatives. Annex 1 in the Supplementary file presents a complete list of the study countries. Of these countries, we drew upon data from Burkina Faso, Ethiopia, and Nigeria for country-level illustration and further analysis to understand within-country variation. We opportunistically selected these three countries in order to align with other analyses included in this Special Issue. Additionally, these countries reflect diverse demographic and economic characteristics and varying levels of family planning programmes especially in recent decades, despite being in the same geographic region (Table 1). Finally, in order to understand the prospects of the 63 countries in an historic context, we analysed estimates data between 1970 and 2010 from all available 194 countries in the database, as described below.

**Table 1:** Selected current population and economic characteristics of Burkina Faso, Ethiopia, and Nigeria

	Burkina Faso	Ethiopia	Nigeria
Total fertility rate	5.9	4.1	5.6
Population (millions)	18	96	178
Rate of natural population increase (%)	3.1	2.1	2.5
Female population 15–49 years of age who have no education (%)^a^	74	51	38
Gross national income per capita, purchasing power parity (US$)	1,560	1,350	5,600

Source: World Population Data Sheet 2014 (Population Reference Bureau), www.prb.org/pdf14/2014-world-population-data-sheet_eng.pdf, unless noted.

a Burkina Faso Demographic and Health Survey (DHS) 2010, Ethiopia DHS 2011, Nigeria DHS 2013.

### Data

Data on country-specific estimates and projections of family planning indicators were from an estimates and projections database of the United Nations Population Division (11). The database includes model-based annual estimates and projections of selected family planning indicators from 1970 to 2030 for 194 countries (www.un.org/en/development/desa/population/publications/dataset/contraception/wcu2014.shtml). The indicators include contraceptive prevalence rate (CPR), modern contraceptive prevalence rate (MCPR), and unmet need for family planning among women between 15 and 49 years of age who are married or in a union. Estimates and projections are based on a Bayesian hierarchical model combined with country-specific time trends, which captures observed fluctuations around the main trends within countries. The modelling data set includes estimates of family planning indicators from a large number of population-based surveys, including country-specific national surveys – some of which were conducted as early as in the 1950s – as well as long-standing global survey programmes such as Demographic and Health Surveys, Multiple Indicator Cluster Surveys, and Reproductive Health Surveys. Detailed methods on the statistical modelling are provided elsewhere (12). For each indicator, the database presents median estimates with 80 and 95% uncertainty bounds. For this study, the median estimate was used for all analyses.

### Measurement and analysis

First, for each of the 194 countries and for the years between 1970 and 2030, we calculated the proposed SDG indicator – percent demand for family planning met with modern contraceptive methods (the ratio of MCPR to the sum of CPR and unmet need for family planning). For the 63 study countries, we then calculated the projected absolute percentage point changes per year between 2014 and 2030 for the indicator. We also calculated the absolute percentage point changes per year between 2014 and 2030 that would be needed to achieve the proposed benchmark – at least 75%, hereinafter referred to as a *benchmark scenario* – using a simple assumption of linear changes in each country. We believe that such a simple calculation is useful for the purpose of this study to provide prospects and facilitate discussion for strategic planning, even though the assumption of linear trends over 15 years was crude, especially for countries currently at a low level. The two annual changes (projected vs. required for the benchmark scenario) were compared by calculating both absolute and relative differences for each country. In addition, we calculated the absolute number of women who would use modern methods in 2030 according to current projections and the benchmark scenario. It should be noted that, under the benchmark scenario, we assume that the currently projected demand trajectory will remain even if MCPR increases rapidly. This assumption ignores a positive relationship between MCPR and demand (12) and may underestimate demand as MCPR accelerates under the benchmark scenario. Thus, our approach may underestimate the required progress as well as the amount of resources needed to respond to increasing demand under the benchmark scenario.

In order to understand the required progress under the benchmark scenario in the context of historic experience, we calculated the annual absolute percent point changes in the proposed SDG indicator over eight 5-year periods (1970–1975, 1975–1980, 1980–1985, 1985–1990, 1990–1995, 1995–2000, 2000–2005, and 2005–2010) using estimates from all 194 countries. We then compared the annual changes in the benchmark scenario for the 63 study countries with these observed, historic annual changes from 194 countries between 1970 and 2010.

Descriptive analyses were conducted using figures and summary statistics, which were unweighted averages across the study countries. To investigate regional patterns, we classified the countries into three groups: Central and West Africa (*n*=22), South and East Africa (*n*=19), and other (*n*=22) (Annex 1 in the Supplementary file). STATA 13.0 statistical software was used for all analyses (Stata Corporation, College Station, TX, USA).

## Results

**Fig. 1:**
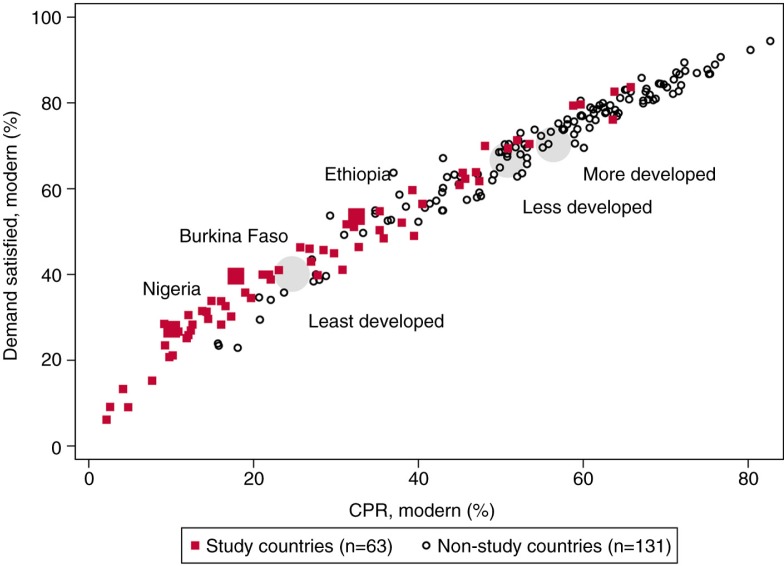
Percentage of demand for family planning met with modern contraceptive methods and modern contraceptive prevalence rates in 194 countries in 2014. Large grey circles represent the unweighted average of countries by development classification by United Nations: least, less, and more developed countries (*n*=49, 103, and 42, respectively).

On average across the 63 study countries in 2014, among women in union, 59% had demand for family planning. Of those women in union who had demand for family planning, on average 43% used modern contraceptive methods, with a large variation among countries ranging from 6% in South Sudan to 84% in Bhutan. Figure 1 presents the current levels of family planning demand satisfied with modern methods and modern contraceptive prevalence among the study countries (in red) and non-study countries (in black). In our three illustrative countries – Nigeria, Burkina Faso, and Ethiopia – 28, 40, and 54% of women who had demand for family planning used modern methods, respectively. Of note, five study countries (Bhutan, Honduras, Indonesia, South Africa, and Zimbabwe) already exceeded the 75% benchmark. Across 49 least developed countries (according to the United Nations classification), on average about 40% of women with demand for family planning used modern methods, compared to 67 and 70% in less and more developed countries. Again, there was great variation across countries, even among the 49 least developed countries, highlighted by the data from five countries (South Sudan, Somalia, Chad, Guinea, and Democratic Republic of Congo) where the level was 15% or below.

Table 2 presents annual changes between 2014 and 2030 according to the current projections and the benchmark scenario in the 63 study countries. Across our study countries, an average annual increase of 2.2 percentage points is needed to achieve the benchmark scenario, compared to the annual increase of 0.9 percentage points in current projections. The average difference between the two rates of annual change is 1.3 percentage points (additional absolute increase) or 2.5 times (relative difference).

**Table 2:** Percentage demand for family planning met with modern methods (2014 and 2030), annual rates of change (2014–2030), and the number of women using modern methods according to current projection and benchmark scenarios among 63 selected countries

	All study countries	West and Central Africa	East and South Africa	Other
Indicator	(*n*=63)	(*n*=22)	(*n*=19)	(*n*=22)
Mean family planning demand satisfied with modern methods (%)^a^				
2014	43.3	27.7	46.7	56.1
2030	56.4	42.4	63.0	64.7
Mean annual rate of change, 2014–2030 (percentage points)^a^ ^b^				
Current projection	0.9	0.9	1.1	0.6
Required by benchmark scenario	2.2	3.0	2.0	1.4
Mean difference in annual rate of changes between benchmark scenario and current projections^a^ ^b^				
Absolute difference (percentage points)	1.3	2.0	0.9	0.8
Relative difference (ratio)	2.5	3.2	1.7	2.3
Total number of women (thousands)^c^				
2014	533,808	71,199	65,419	397,190
2030	615,063	96,341	92,889	425,833
Total number of women using modern methods (thousands)^c^				
2014	225,841	7,989	21,564	196,289
2030, current projection	300,629	21,214	45,431	233,984
2030, benchmark scenario^d^	333,744	39,068	50,994	243,683

a Mean is unweighted average of national-level values across countries;

b excluding five countries where family planning demand satisfied with modern methods in 2014 was 75% or above;

c total number of women is aggregate sum across countries (*women* refers to those who are between 15 and 49 years of age and married or in a union);

d nine countries where projection is 75% or higher, the number of women according to the projected level was used.

**Fig. 2:**
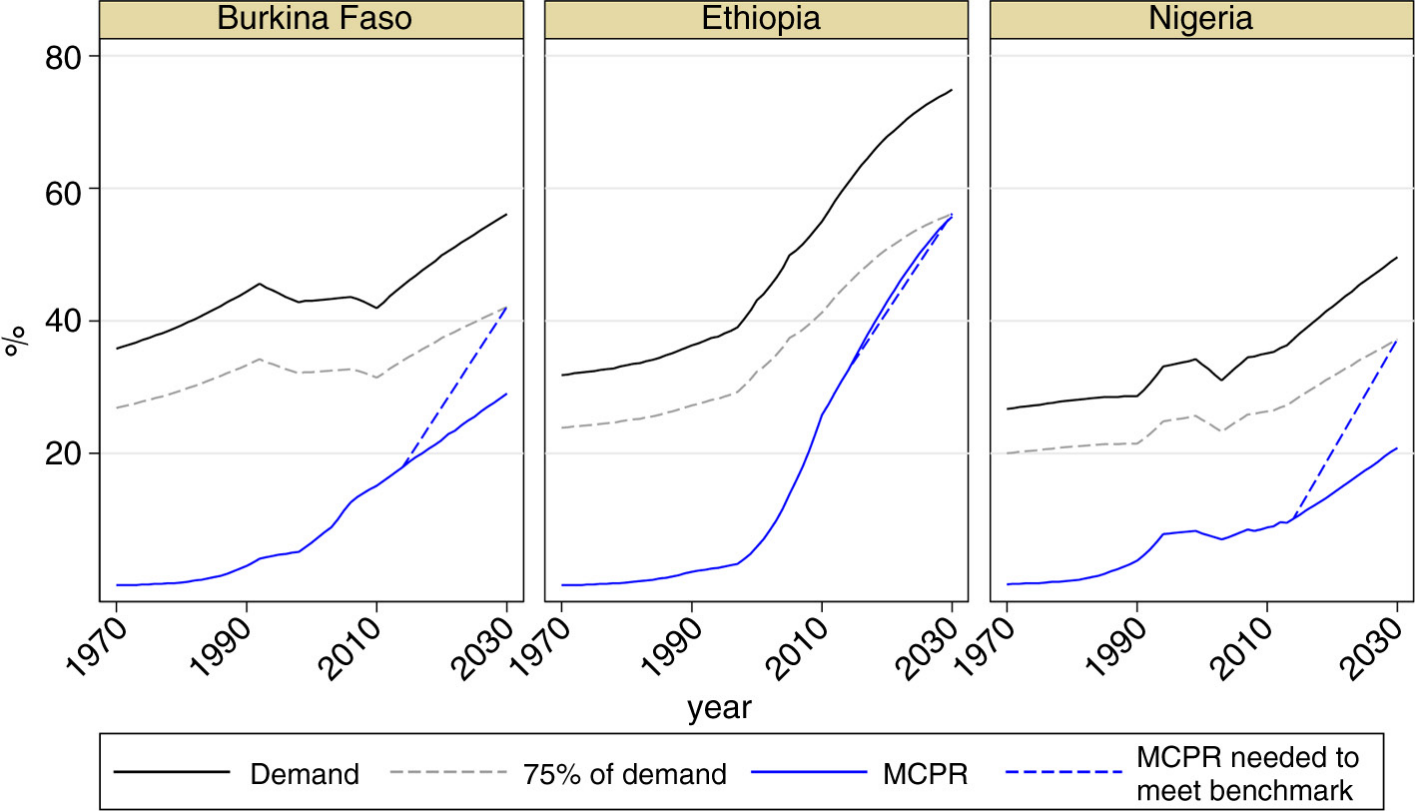
Demand for family planning and modern contraceptive prevalence rates in Burkina Faso, Ethiopia, and Nigeria: current estimates and projections versus progress needed to meet the benchmark of 75% by 2030. MCPR, modern contraceptive prevalence rate.

Figure 2 illustrates differences in progress between the current projection (solid blue line) and benchmark scenario (dotted blue line) in Burkina Faso, Ethiopia, and Nigeria. In Ethiopia, the MCPR is projected to be 56% in 2030, which equates to 75% of family planning demand. Ethiopia shows little difference between the projected and required annual rates of change (Table 3). If its currently projected progress is realised, Ethiopia will achieve the family planning component of SDGs. In contrast, current projections for Burkina Faso and Nigeria indicate that only 52 and 42% of demand for family planning, respectively, will be satisfied with modern method use by 2030. Differences between projections and the benchmark scenario in these two countries are substantial (Table 3). To reach the benchmark, Burkina Faso must have an average yearly growth of 2.2 percentage points, and Nigeria must reach an average annual growth of 3.0 percentage points. Both rates of growth are much higher than those based on current projections – 0.8 and 0.9 percentage point increases per year for Burkina Faso and Nigeria, respectively.

**Table 3:** Percentage of demand for family planning met with modern methods (2014 and 2030), annual rates of change (2014–2030), and the number of women using modern methods according to current projection and benchmark scenarios in Burkina Faso, Ethiopia, and Nigeria

Indicator	Burkina Faso	Ethiopia	Nigeria
Mean family planning demand satisfied with modern methods (%)			
2014	39.5	53.5	27.2
2030	51.7	74.4	41.9
Mean annual rate of change, 2014–2030 (percentage points)			
Current projection	0.8	1.3	0.9
Required by benchmark scenario	2.2	1.3	3.0
Difference in annual rate of change between the benchmark scenario and current projections			
Absolute difference (percentage points)	1.5	0.0	2.1
Relative difference (ratio)	2.9	1.0	3.2
Total number of women (thousands)^a^			
2014	3,078	13,935	26,645
2030	4,224	20,588	34,604
Total number of women using modern methods (thousands)^a^			
2014	551	4,529	2,691
2030, current projection	1,225	11,468	7,198
2030, benchmark scenario	1,777	11,565	12,873

a *Women* refers to those who are between 15 and 49 years of age and married or in a union.

As illustrated by these three country examples, vastly different prospects for achieving the benchmark are observed across all study countries. Differences between the current projection and benchmark scenario paths are largest in the Central and West African countries (*n*=22), where annual changes in the benchmark scenario were on average 3.2 times higher than those changes according to current projections (Table 2). In contrast, differences between the current projection and benchmark scenario are substantially smaller in the South and East African countries (*n*=19), where the average annual change required to meet the benchmark scenario is 1.7 times higher than current projections. In about one-quarter of our study countries, the current projection for 2030 is at or near the benchmark. Specifically, the current projection for 2030 is 75% or above in nine countries and is near the benchmark (i.e. 70–75%) in six countries (Annex 2 in Supplementary file).

**Fig. 3:**
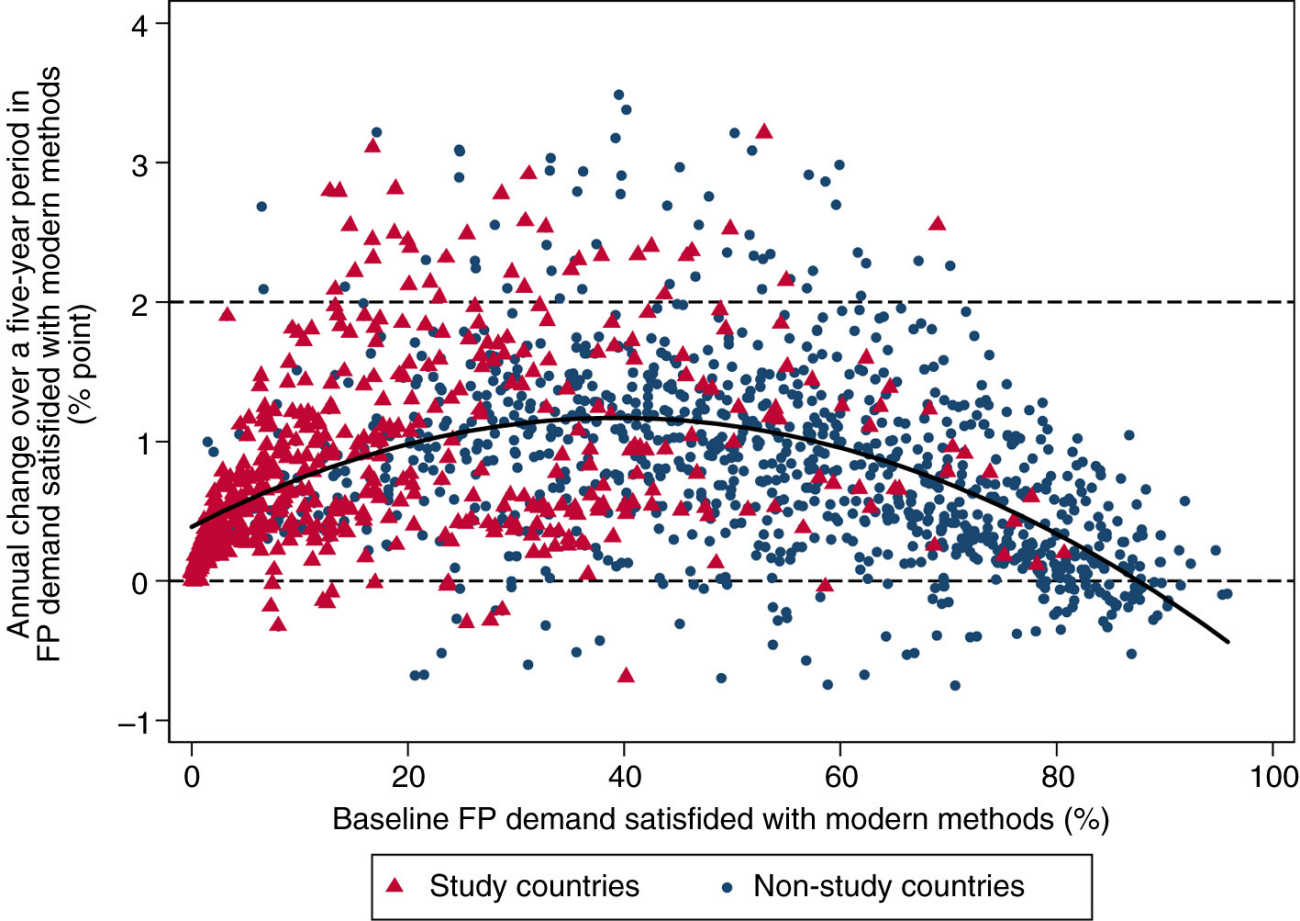
Scatter plot of annual rate of changes in demand for family planning satisfied with modern methods by initial level: eight 5-year periods between 1970 and 2010 from 194 countries. Solid curved line is a quadratic fitted line (*R* ^2^ 0.17). A total of 1,552 observations are specific to a 5-year period and a country. The annual percentage point changes range from −3.87 (Timor-Leste 1995–2000) to 7.81 (Rwanda 2005–2010). Only 1,520 observations between the 1st and 99th percentile of the changes are shown, ranging from −0.78 to 3.50.

Historic trends indicate that the progress required to achieve the benchmark will be challenging for the majority of the study countries. Figure 3 is a scatter plot of annual percentage point changes that were observed from 194 countries over each of the eight 5-year periods spanning the years 1970–2010. In the overwhelming majority of cases (94%), the annual change was below 2 percentage points. Further, the annual changes appeared to be higher when the initial level of demand satisfied was between 30 and 50%, as indicated by the quadratic fitted line. An annual increase of 2 percentage points or higher was observed at least once in 24 study countries and 37 non-study countries. Of the 24 study countries, 2 were in Central and West Africa (Burkina Faso, from 15% in 2000 to 26% in 2005, and Nigeria, from 13% in 1990 to 24% in 1995); 12, including Ethiopia, were in South and East Africa; and 10 were outside Africa (Table 4). Nevertheless, in most of these countries, the rapid increase was observed over only 5- or 10-year periods, suggesting that maintaining the momentum over 15 years will be challenging. When we examined average rates of change over rolling 15-year periods between 1970 and 2010, only 13 study countries experienced annual average changes by 2 percentage points or more: Bangladesh, Bhutan, Cambodia, Ethiopia, India, Indonesia, Madagascar, Malawi, Mozambique, Myanmar, Rwanda, South Africa, and Zimbabwe (results not shown).

**Table 4:** List of countries and periods during which the annual change in family planning demand satisfied with modern methods exceeded two percentage points

		Demand for family planning satisfied with modern methods (%)		Annual rate of change (% points)
		
Country	Period	Beginning	Ending	
Afghanistan	2000–2005	14.7	27.4	2.5
Bangladesh	1980–1985	23.6	35.1	2.3
Bangladesh	1985–1990	35.1	46.3	2.2
Bangladesh	1990–1995	46.3	58.1	2.4
Bhutan	1990–1995	30.8	41.3	2.1
Bhutan	1995–2000	41.3	53.0	2.3
Bhutan	2000–2005	53.0	69.0	3.2
Bhutan	2005–2010	69.0	81.8	2.5
Burkina Faso	2000–2005	15.1	26.2	2.2
Burundi	2005–2010	20.3	32.2	2.4
Cambodia	1995–2000	18.9	32.9	2.8
Djibouti	2000–2005	12.8	26.8	2.8
Ethiopia	2000–2005	13.7	27.7	2.8
Ethiopia	2005–2010	27.7	46.9	3.9
India	1970–1975	22.1	32.8	2.1
India	1975–1980	32.8	45.4	2.5
Indonesia	1970–1975	16.8	32.3	3.1
Indonesia	1975–1980	32.3	49.8	3.5
Indonesia	1980–1985	49.8	62.4	2.5
Kenya	1985–1990	20.0	32.2	2.4
Laos	1995–2000	35.8	47.3	2.3
Lesotho	1985–1990	20.1	30.7	2.1
Lesotho	2005–2010	55.0	65.8	2.2
Madagascar	2000–2005	25.5	37.9	2.5
Madagascar	2005–2010	37.9	49.6	2.3
Malawi	1995–2000	23.1	40.8	3.5
Mozambique	1995–2000	16.7	28.9	2.4
Myanmar	1990–1995	30.9	43.8	2.6
Myanmar	1995–2000	43.8	54.0	2.1
Nepal	1985–1990	22.9	33.1	2.0
Nigeria	1990–1995	13.3	23.7	2.1
Rwanda	2005–2010	18.4	57.4	7.8
South Africa	1970–1975	28.7	42.5	2.8
South Africa	1975–1980	42.5	54.5	2.4
Timor-Leste	2005–2010	29.6	40.7	2.2
Tanzania	1990–1995	16.8	28.4	2.3
Zimbabwe	1975–1980	18.8	31.2	2.5
Zimbabwe	1980–1985	31.2	45.8	2.9
Zimbabwe	1985–1990	45.8	57.4	2.3

An annual increase of three percentage points or more – the average level of progress needed among the Central and West African countries – was observed over a 5-year period in five study countries (Bhutan, Ethiopia, Indonesia, Malawi, and Rwanda) (Table 4) and 14 non-study countries. However, when we examined the rapid increase sustained over a 15-year period, only six countries in the world experienced annual average changes by 3 percentage points or more over 15 years or longer during the last four decades: Belgium (from 24% in 1970 to 82% in 1987), Botswana (from 3% in 1972 to 56% in 1992), Colombia (from 20% in 1970 to 65% in 1985), France (from 23% in 1970 to 77% in 1987), Indonesia (from 17% in 1970 to 69% in 1988), and Réunion (from 25% in 1970 to 76% in 1987).

Figure 3 also shows that some countries experienced negative changes (7% of total observation points). Whereas most of the negative changes were less than 1 percentage point per year, substantial reductions were often observed in the wake of serious turmoil, civil unrest, and war. These examples include Timor-Leste (from 53% in 1995 to 34% in 2000), Serbia (from 38% in 2000 to 28% in 2005), Mozambique (from 37% in 2005 to 30% in 2010), and Rwanda (from 15% in 1995 to 9% in 2000).

Lastly, we examined the absolute number of women who would use modern methods in 2030 under current projections and the benchmark scenario based on 1) population projections of women of reproductive age who are in a union; 2) demand for family planning according to current projections; and 3) demand for family planning satisfied with modern methods according to both current projections and the benchmark scenario. The examples of Burkina Faso, Ethiopia, and Nigeria (Table 3) show that the number of women who would use modern methods in 2030 based on current projections is 1.2 million, 11.5 million, and 7 million, respectively. As expected, the difference in the number of women using modern methods in 2030 between the current projection and the benchmark scenario is minimal in Ethiopia, but substantial in Burkina Faso and Nigeria: The absolute number of women who would be using modern methods was 45 and 79% higher in the benchmark scenario than the current projection, respectively. Throughout all 63 study countries, 300 million and 334 million women would be using modern methods in 2030, according to the current projection and benchmark scenario, respectively (Table 2). In 22 Central and West African countries, almost twice as many as women would be using modern methods under the benchmark scenario (39 million) compared to the current projection (21 million) (Table 2).

## Discussion

Our study examines the prospects of 63 selected countries towards achieving the family planning indicator and benchmark for the SDG target of universal access to sexual and reproductive health services. There is great variation across the study countries, but on average, a sustained increase of 2.2 percentage points per year is required over the next 15 years. Compared to current projections, the rates of change in the 63 study countries need to double in order to achieve the benchmark. In the Central and West Africa regions, progress needs to almost triple.

Examination of historic rates of progress shows that rapid annual increases by 2 percentage points or higher over 5 years have been observed in 24 of the 63 study countries. However, sustaining the rapid progress over an extended period is challenging, as shown by the fact that only 13 study countries achieved rapid progress by two percentage points or more per year over a 15-year period. Examples of annual increases by three percentage points or more over 15 years were rare. Further, only five study countries experienced an annual change of three percentage points or higher over a 5-year period and only one study country over a 10-year period. Nevertheless, examples from non-study countries, especially Botswana, where a rapid and sustained progress was made even with a very low initial level, suggest that the benchmark may be achievable.

Among our three illustrative countries, Ethiopia made remarkably rapid improvements between 2000 and 2010, thanks to political will and strategic programming (13, 14). Current projections suggest that Ethiopia is well on its way to meeting the benchmark by 2030. In Burkina Faso and Nigeria, two things should be noted. First, currently projected demand is relatively low, and the countries can achieve the benchmark of 75% with a fairly low level of MCPR. In countries that are in the beginning of fertility transition, implementing multisectoral approaches to generate demand for family planning is critical, in addition to efforts to satisfy current demand. Secondly, even with the relatively low demand that is projected currently, the two countries need to accelerate the rate of progress by about three times to meet the benchmark. Burkina Faso and Nigeria have had rapid increases historically, though only over one 5-year period each (Table 4). This historic experience suggests that, though ambitious, the benchmark can be achieved even in countries with currently low levels of demand satisfied with modern methods.

Nevertheless, there are substantial challenges to achieving the family planning benchmark in many countries. Simply to achieve current projections, which are based on assumptions of continuous and universal increases in modern contraceptive use across countries, will require continuous and intensive programme efforts. Historic data show that stagnation or even significant reversal can be observed in some countries under humanitarian crises. Thus, in order to accelerate currently projected rates of improvement, even greater amounts of resources, both financial and technical, and more efficient use of resources will be needed to stimulate demand for family planning and improve access. Indeed, a recent study showed financial resources need to almost double to fully address all current needs for sexual and reproductive health services, including modern contraception, in developing countries (15).

**Fig. 4:**
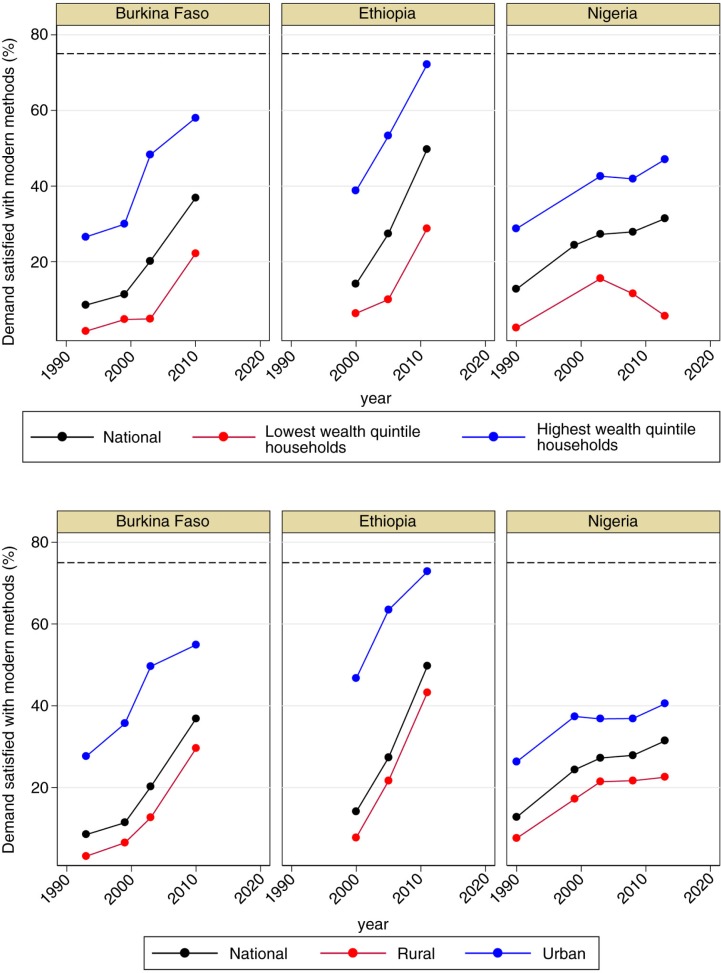
Trends in demand satisfied with modern methods by household wealth and residential area: Burkina Faso, Ethiopia, and Nigeria, by socio-economic and rural–urban status. Source: Demographic and Health Surveys through STATcompiler.com.

While our study focuses on national-level prospects, an important factor for achieving the ‘universal access’ component of the SDGs is addressing inequalities within each country. Further examination of data from Burkina Faso, Ethiopia, and Nigeria reveals within-country inequality (Fig. 4). Disaggregated data by household wealth quintile and residential area are presented for illustrative purposes across the three countries, although monitoring disaggregated data by other characteristics such as administrative unit – especially in the context of programming and/or decentralisation – is imperative. Some subpopulations such as women living in urban areas or highest wealth quintile households have made strong progress. In Ethiopia, especially, family planning demand satisfied with modern methods is already near the SDG benchmark among women in the highest wealth quintile households. In contrast, progress in disadvantaged populations is much slower. Further, in Nigeria, family planning demand satisfied with modern methods decreased among women in the lowest wealth quintile households during the last decade, although the national average increased steadily. At a more global scale, a recent study using data from 187 Demographic Health Surveys conducted in 53 countries showed that national average and within-country disparity are inversely related; as national average for demand satisfied with modern methods increases, within-country disparity decreases (16). The study also reported that within-country disparity by socio-economic background has decreased in the last two decades in most countries. Recognising the imperative of the SDGs to leave no one behind, efforts to satisfy demand for family planning must focus on bridging the inequality gaps across subpopulations.

One limitation of our study is that we used an estimates and projections database that included only women who were currently married or in a union. Omission of unmarried, sexually active women in the database is the consequence of current inconsistent data availability among unmarried women at the global scale, not because of lack of importance of the population. Age at marriage has increased in many countries around the world, and there are a growing number of unmarried, sexually active women who have demand for family planning. Further, all women, regardless of their marital status – or any other characteristic such as young age – must have full and equal access to appropriate information and quality contraception services in order to be able to achieve their reproductive intentions to delay, space, or limit pregnancy. Thus, to align the indicator with the intentions of the goal and benchmark, the global database should include unmarried, sexually active women. That being said, the impact of these data on the estimate may not be substantial because this is a small population.

The data and findings presented provide baseline information to identify strategies needed at both global and country levels to achieve the family planning component of the SDGs. For example, our findings foremost show a great need to increase efforts and improve efficiency of investment in order to achieve the family planning component of the SDGs. Our identification of countries that have achieved rapid and sustained progress provides the geography of family planning policy and programmatic successes to explore and replicate as appropriate (13, 14, 17, 18). Historic examination also suggests progress may accelerate once populations reach a certain level, but until that point great efforts would be needed both to generate demand and to address existing demand. Finally, our identification of countries that have experienced declines in satisfied demand – though a small number – highlights the negative impacts conflicts and wars can have in relation to access to sexual and reproductive health services. This historic information is valuable to the international family planning community as evidence of the need to mitigate the detrimental impacts of existing and future tumult on family planning access.

Substantial variations in current levels and future prospects for demand satisfied among study countries raises questions for international and bilateral development organisations as well as countries. For example, how will both global and domestic financial and technical resources be mobilised and used over the next 15 years to achieve maximal outcomes and impact? How should development partners prioritise focal countries considering vastly different prospects even among the 63 study countries? What are approaches for monitoring at the global level, balancing achievement of the benchmark in each country versus reaching the largest absolute number of potential beneficiaries in highly populous countries?

## Conclusions

On average across the 63 study countries, family planning demand satisfied with modern contraceptive methods needs to increase by 2.2 percentage points per year for the next 15 years – more than double current projections. Yet historic experience during the last four decades shows that such rapid progress has been very infrequently achieved. More financial and technical resources and more efficient use of them, including a regional focus on Central and West Africa, are therefore critical to enabling countries to accelerate progress and achieve the ambitious benchmark scenario of at least 75% of family planning demand being satisfied with modern methods.

## Supplementary Material

Meeting demand for family planning within a generation: prospects and implications at country level
