# Supplementary material for: Meeting demand for family planning within a generation: prospects and implications at country level
Source: Glob Health Action. 2015 Nov 9;8:10.3402/gha.v8.29734. doi: 10.3402/gha.v8.29734 (PMC4642369; doi:10.3402/gha.v8.29734)
Supplement: Meeting demand for family planning within a generation: prospects and implications at country level [file GHA-8-29734-s001.pdf]

Supplementary File to Choi et al. "Meeting demand for family planning within a generation: Prospects and implications at country level", *Global Health Action* 2015; 8:29734.

Annex 1. List of 63 selected study countries

Country	Least developed [a]	Priority for international initiatives or programme				Region
		Ouagadougou partnership	FP2020 pledging	USAID priority [b]	UNFPA priority [c]	
Afghanistan	Yes			Yes		Asia
Angola	Yes					Central and West Africa
Bangladesh	Yes		Yes	Yes		Asia
Benin	Yes	Yes			Yes	Central and West Africa
Bhutan	Yes					Asia
Bolivia (Plurinational State of)					Yes	Latin America
Burkina Faso	Yes	Yes	Yes		Yes	Central and West Africa
Burundi	Yes				Yes	South and East Africa
Cambodia	Yes					Asia
Cameroon					Yes	Central and West Africa
Central African Republic	Yes				Yes	Central and West Africa
Chad	Yes				Yes	Central and West Africa
Comoros	Yes					South and East Africa
Cote d'Ivoire		Yes	Yes		Yes	Central and West Africa
Democratic Republic of the Congo	Yes		Yes	Yes	Yes	Central and West Africa
Djibouti	Yes				Yes	South and East Africa
Equatorial Guinea	Yes					Central and West Africa
Eritrea	Yes				Yes	South and East Africa
Ethiopia	Yes		Yes	Yes	Yes	South and East Africa
Gambia	Yes				Yes	Central and West Africa
Ghana			Yes	Yes	Yes	Central and West Africa
Guinea	Yes	Yes			Yes	Central and West Africa
Guinea-Bissau	Yes				Yes	Central and West Africa
Haiti	Yes			Yes	Yes	Latin America
Honduras					Yes	Latin America
India			Yes	Yes		Asia
Indonesia			Yes			Asia
Kenya			Yes	Yes	Yes	South and East Africa
Kiribati	Yes					Oceania
Lao People's Democratic Republic	Yes				Yes	Asia
Lesotho	Yes				Yes	South and East Africa
Liberia	Yes			Yes	Yes	Central and West Africa
Madagascar	Yes			Yes	Yes	South and East Africa
Malawi	Yes		Yes	Yes	Yes	South and East Africa
Mali	Yes	Yes		Yes	Yes	Central and West Africa
Mauritania	Yes	Yes			Yes	Central and West Africa
Mozambique	Yes		Yes	Yes	Yes	South and East Africa
Myanmar	Yes				Yes	Asia

Nepal	Yes			Yes	Yes	Asia
Niger	Yes	Yes	Yes		Yes	Central and West Africa
Nigeria			Yes	Yes	Yes	Central and West Africa
Pakistan			Yes	Yes		Asia
Papua New Guinea					Yes	Oceania
Philippines			Yes	Yes		Asia
Rwanda	Yes		Yes	Yes	Yes	South and East Africa
Samoa	Yes					Oceania
Sao Tome and Principe	Yes				Yes	Central and West Africa
Senegal	Yes	Yes	Yes	Yes	Yes	Central and West Africa
Sierra Leone	Yes		Yes		Yes	Central and West Africa
Solomon Islands	Yes		Yes			Oceania
Somalia	Yes					South and East Africa
South Africa			Yes			South and East Africa
South Sudan	Yes			Yes	Yes	South and East Africa
Sudan	Yes				Yes	South and East Africa
Timor-Leste	Yes				Yes	Asia
Togo	Yes	Yes			Yes	Central and West Africa
Tuvalu	Yes					Oceania
Uganda	Yes		Yes	Yes	Yes	South and East Africa
United Republic of Tanzania	Yes			Yes	Yes	South and East Africa
Vanuatu	Yes					Oceania
Yemen	Yes			Yes	Yes	Asia
Zambia	Yes		Yes	Yes	Yes	South and East Africa
Zimbabwe			Yes		Yes	South and East Africa

a. Least developed according to classification by United Nations.

b. Priority countries for the Office of Population and Reproductive Health, Bureau for Global Health.

c. Priority countries for the Global Program on Reproductive Health Commodity Security.

Note: There is no data for Congo Brazzaville in the UN database, and thus the country was excluded from the study. There are 69 countries under FP2020, but the study only includes countries where the government made an official pledge.

Annex 2. Demand for family planning, modern contraceptive prevalence rate, demand satisfied with modern methods in 2030, according to current projections by United Nations: 63 study countries.

Country*	Demand for family planning (%)	Modern contraceptive prevalence rate (%)	Demand for family planning satisfied with modern methods (%)
Bhutan	81.6	71.1	87.1
South Africa	79.2	66.7	84.2
Zimbabwe	78.0	64.3	82.4
Indonesia	75.9	60.7	80.0
Lesotho	77.9	60.6	77.8
Honduras	83.4	64.4	77.2
Myanmar	74.8	57.6	77.0
Malawi	78.7	60.1	76.4
India	76.1	57.1	75.0
Rwanda	78.5	58.7	74.8
Ethiopia	74.9	55.7	74.4
Bangladesh	78.6	57.9	73.7
Nepal	79.4	57.6	72.5
Lao People's Democratic Republic	76.8	55.6	72.4
Kenya	78.4	55.9	71.3
Madagascar	73.4	50.6	68.9
Cambodia	78.3	52.2	66.7
United Republic of Tanzania	72.0	47.8	66.4
Zambia	75.9	49.9	65.7
Uganda	74.6	48.3	64.7
Vanuatu	69.2	44.6	64.5
Timor-Leste	65.3	40.8	62.5
Djibouti	67.4	42.1	62.5
Philippines	76.0	45.5	59.9
Burundi	68.2	40.8	59.8
Bolivia (Plurinational State of)	81.6	48.5	59.4
Pakistan	69.9	41.5	59.4
Sao Tome and Principe	75.4	44.6	59.2
Haiti	75.3	44.1	58.6
Afghanistan	69.2	40.4	58.4
Solomon Islands	65.9	38.4	58.3
Mozambique	60.5	33.1	54.7
Yemen	77.1	41.8	54.2
Papua New Guinea	67.0	36.0	53.7
Samoa	75.7	39.7	52.4
Burkina Faso	56.1	29.0	51.7
Tuvalu	68.5	35.3	51.5
Eritrea	63.5	32.0	50.4
Kiribati	62.5	31.3	50.1

Liberia	60.9	29.7	48.8
Ghana	63.6	30.4	47.8
Comoros	67.5	32.1	47.6
Cameroon	63.2	30.0	47.5
Sierra Leone	57.0	26.5	46.5
Cote d'Ivoire	56.8	26.4	46.5
Senegal	58.0	26.9	46.4
Togo	64.8	29.7	45.8
Guinea-Bissau	52.5	23.8	45.3
Sudan	58.0	26.2	45.2
Mauritania	57.8	25.8	44.6
Niger	47.5	20.4	42.9
Mali	51.5	21.6	41.9
Nigeria	49.6	20.8	41.9
Somalia	67.3	28.0	41.6
Central African Republic	57.7	23.5	40.7
Angola	58.8	23.6	40.1
Benin	56.4	22.1	39.2
Gambia	50.6	19.3	38.1
Equatorial Guinea	58.3	20.6	35.3
DRC	60.1	18.0	30.0
South Sudan	53.5	15.5	29.0
Guinea	44.0	12.5	28.4
Chad	40.5	9.3	23.0

*Countries are presented in the descending order of demand satisfied with modern methods.
